# The role of inflammation in neurodegeneration: novel insights into the role of the immune system in *C9orf72* HRE-mediated ALS/FTD

**DOI:** 10.1186/s13024-022-00525-z

**Published:** 2022-03-18

**Authors:** Pegah Masrori, Jimmy Beckers, Helena Gossye, Philip Van Damme

**Affiliations:** 1 Department of Neurosciences, Experimental Neurology, and Leuven Brain Institute (LBI), KU Leuven-University of Leuven, 3000 Leuven, Belgium; 2 Laboratory of Neurobiology, Experimental Neurology, Center for Brain and Disease Research, VIB, Campus Gasthuisberg, O&N5, Herestraat 49, 602, 3000 Leuven, PB Belgium; 3 Neurology Department, University Hospitals Leuven, Campus Gasthuisberg, Herestraat 49, 3000 Leuven, Belgium; 4 Department of Neurology, University Hospital Antwerp, 2650 Edegem, Belgium; 5 VIB Center for Molecular Neurology, Neurodegenerative Brain Diseases, University of Antwerp, 2000 Antwerp, Belgium; 6 Department of Biomedical Sciences, University of Antwerp, 2000 Antwerp, Belgium

**Keywords:** *C9orf72* HRE-mediated ALS/FTD, Neuroinflammation, Microglia, Astrocytes, Peripheral immune cells, In-vivo and ex-vivo models, Anti-inflammatory therapies

## Abstract

Neuroinflammation is an important hallmark of amyotrophic lateral sclerosis (ALS) and frontotemporal lobar degeneration (FTLD). An inflammatory reaction to neuronal injury is deemed vital for neuronal health and homeostasis. However, a continued activation of the inflammatory response can be detrimental to remaining neurons and aggravate the disease process. Apart from a disease modifying role, some evidence suggests that neuroinflammation may also contribute to the upstream cause of the disease. In this review, we will first focus on the role of neuroinflammation in the pathogenesis of chromosome 9 open reading frame 72 gene (*C9orf72*) hexanucleotide repeat expansions (HRE)-mediated ALS/FTD (C9-ALS/FTD). Additionally, we will discuss evidence from ex vivo and in vivo studies and finally, we briefly summarize the trials and progress of anti-inflammatory therapies.

## Background

Amyotrophic lateral sclerosis (ALS) and frontotemporal lobar degeneration (FTLD) are adult-onset progressive neurodegenerative diseases that are part of the same disease spectrum [1]. Both diseases are characterized by the accumulation of neurotoxic protein depositions, consisting of TAR DNA binding protein (TDP-43) in > 95% of ALS cases and about 50% of FTLD cases [2, 3]. The neuroanatomical distribution of neuropathological lesions varies and correlates with the clinical presentation. In ALS, the motor system is primarily affected with degeneration of motor neurons (MNs) in the motor cortex, brainstem and spinal cord, while in FTLD, the frontal and anterior temporal cortex are mostly affected. The clinical phenotype of ALS is characterized by a combination of upper and lower motor involvement leading to muscle weakness, hyperreflexia, spasticity, as well as muscle atrophy and fasciculations. The disease typically has a focal onset, most commonly with unilateral distal weakness in one limb or tongue weakness, but has a tendency to spread to other body regions [4–7]. FTLD patientspresent with a clinical syndrome of frontotemporal dementia (FTD), a subtype of dementia recognizable primarily by either changes in behaviour (behavioral variant FTD (bvFTD)) and deficits in language (primary progressive aphasia (PPA)), or both [8–10]. In approximately 10% of ALS cases and 40% of FTD cases, the family history is positive and such patients are classified as familial ALS (fALS) or familial FTD (fFTD), respectively. It is mainly transmitted via an autosomal dominant Mendelian inheritance pattern. The remaining cases are classified as sporadic disease (sALS or sFTD) [7]. Significant overlap exists between ALS and FTD, as 10–15% of ALS patients also develop FTD and a similar proportion of FTD patients also develop motor problems during the course of their disease [1, 4, 11–13]. A common genetic basis also exists. The most common cause of both fALS (40%) and fFTD (25%) [14, 15] are GGGGCC hexanucleotide repeat expansions (HRE) in the 5’ non-coding region of *chromosome 9 open reading frame 72* gene (*C9orf72*) [14–16], which will be the focus of this review. How *C9orf72* HRE cause neurodegeneration is only partially understood and several disease mechanisms have been put forward. The neurodegenerative process is accompanied by neuroinflammation, similar to what is observed in other neurodegenerative diseases [17]. Such an inflammatory reaction to neuronal damage is deemed to be important and may contribute to the disease pathogenesis. In this review, our aim is to characterize the role of neuroinflammation in the pathogenesis of C9-ALS/FTD and relate this to the different disease mechanisms proposed for these diseases.

## *C9orf72*-mediated ALS/FTD

Mutations in the *C9orf72* gene are inherited as an autosomal dominant disease with incomplete penetrance [18, 19]. Some *C9orf72* HREs carriers (C9-carriers) develop the disease at an early age while in other carriers the disease doesn’t manifest before their ninth decade of life. By the age of 58, about 50% of carriers will have ALS or FTD [18].

The exact number of repeats sufficient to cause C9-ALS/FTD has not been strictly determined, and consequently, more studies are needed. Currently, a repeat number of 30 is regularly used as threshold. This makes sense, since most patients have hundreds to thousands of repeats. Nonetheless, some reports of ALS and FTD cases with only 20–30 repeats exist [19–25] and it has also been suggested that carrying an intermediate expansion on both alleles might contribute to ALS [26].

Interestingly, repeat sizes can vary between family members with C9-ALS/FTD, even between monozygotic twins [24, 27]. On top of that, the instability of the expansion also causes a variation in repeat sizes of the expansion between different tissues within the same individual. This way, the repeat size measured in blood may differ from the repeat size present in brain [23, 28–30]. A correlation between repeat size and age of onset, disease progression rate and survival has been suggested, but remains unclear [24, 31–33]. The association between repeat size and age of onset is potentially biased by the confounding factor of age at sample collection [24].

## Pathological characterization of C9-ALS/FTD

Post-mortem brain and spinal cord tissues of C9-ALS/FTD patients revealed some highly characteristic pathological features in addition to the TDP-43 pathological lesions, including RNA foci and protein aggregates containing dipeptide repeats (DPRs) and the autophagy adaptor protein p62/sequestosome1 (p62) [34, 35].

The presence of RNA foci, consisting of sense and anti-sense G4C2 repeat RNA, the spliced intron 1 of the gene, or the complete *C9orf72* (pre-)messenger RNA (mRNA), has been described in C9-ALS/FTD brain and spinal cord tissues [14, 27, 36]. These RNA foci are predominantly localized in neuronal nuclei in the frontal and motor cortices, hippocampus, cerebellum and in the spinal cord [37]. They have also been found in induced pluripotent stem cell (iPSC)-derived motor and cortical neurons, fibroblasts and sometimes also in patient-derived astrocytes, microglia and oligodendrocytes [38–40].

Another pathologic hallmark of C9-ALS/FTD is the presence of toxic DPRs. These are produced by repeat-associated non-AUG (RAN) translation of *C9orf72* repeat RNA. Poly-glycine-alanine (GA) and poly-glycine-arginine (GR) DPR species are translated from sense G4C2 transcripts, while poly-proline-alanine (PA) and poly-proline-arginine (PR) proteins originate from antisense G4C2 transcripts, and poly-glycine-proline (GP) proteins may originate from both sense and antisense transcripts [36, 38, 41–43]. In C9-ALS/FTD, DPR inclusions are present in the cytoplasm and nucleus of neurons, mostly in hippocampus, thalamus, cortex, amygdala and the cerebellar granular layer [21, 42, 44]. These DPR-inclusions mostly stain positive for ubiquitin and p62 while they do not colocalize with TDP-43 inclusions [21].

C9-ALS/FTD is associated with TDP-43 inclusions, as is common in non-C9-ALS/FTD as well. TDP-43 pathology can be found in about 97% of ALS patients and in about 50% of FTD. [45, 46]. Recently, a study showed that poly-GR may directly sequester TDP-43 and induce TDP-43 aggregation [47]. The cytoplasmic and/or intranuclear pTDP-43 inclusions in C9-carriers are accompanied by nuclear depletion of TDP-43 and can be found in the cerebral cortex and subcortical regions such as the striatum, hippocampus, basal ganglia, and substantia nigra [48–52]. Some studies also reported cytoplasmic p62 and pTDP-43-positive inclusions in glial cells, such oligodendrocytes and astrocytes [35, 53].

## Pathomechanism of C9-ALS/FTD

**Fig. 1:**
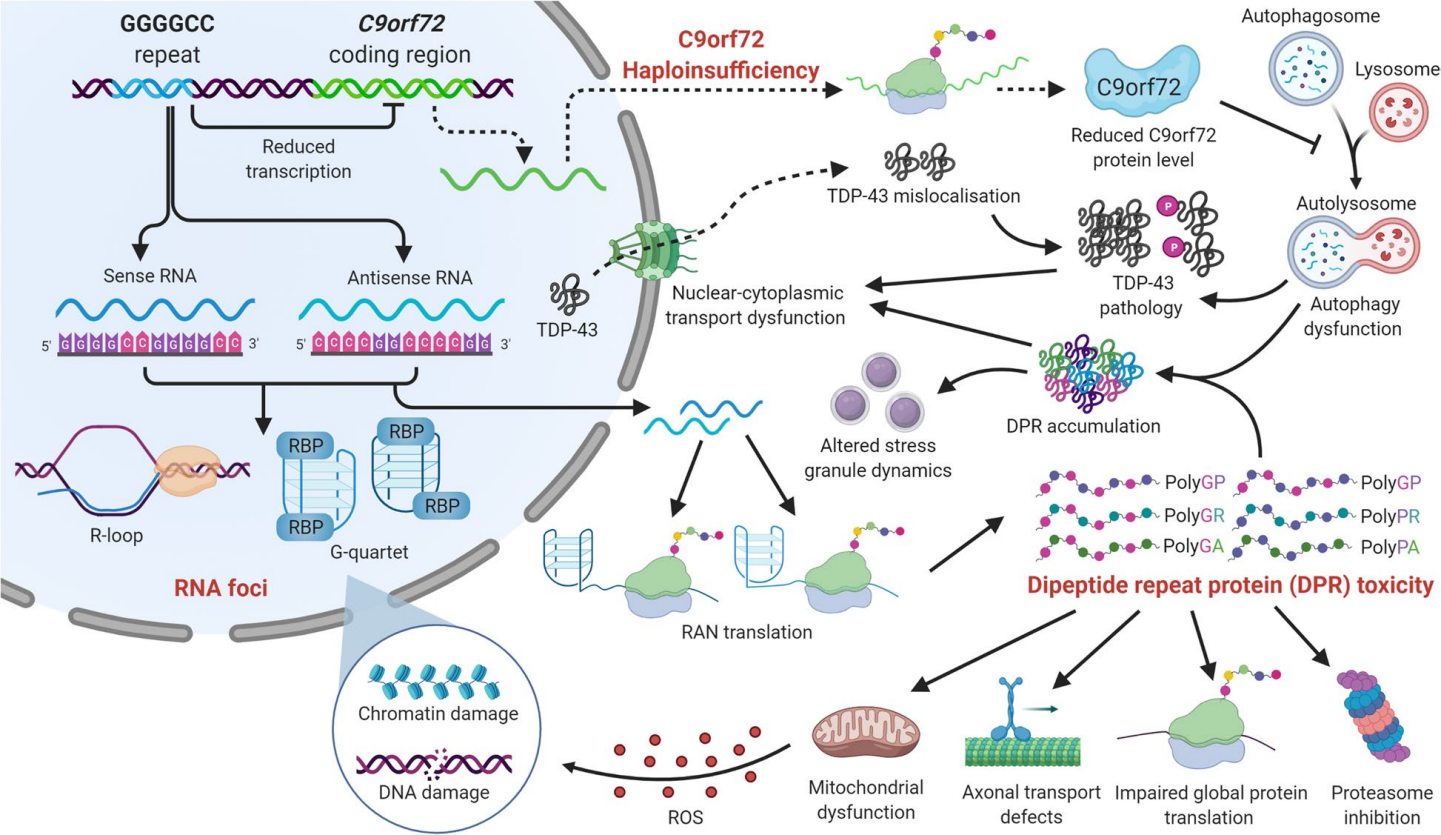
Overview of the different disease mechanisms at play in C9orf72-ALS/FTD. First, the (GGGGCC)n hexanucleotide repeat expansion could reduce transcription of the *C9orf72* gene leading to reduced C9orf72 protein levels (haploinsufficiency) that may ultimately cause dysfunction of the autophagy-lysosome pathway. Second, bidirectional transcription of the repeat expansion forms sense (GGGGCC) and antisense (CCCCGG) RNA transcripts that form secondary structures and may cause RNA toxicity by sequestering essential RNA-binding proteins (RBPs). Finally, unconventional repeat-associated non-ATG (RAN) translation produces toxic dipeptide repeat proteins (DPRs): polyGP, polyGR and polyGA from the sense strand and polyGP, polyPR and polyPA from the antisense strand. These toxic DPRs are able to form aggregates and may affect several essential cellular pathways such as mitochondrial function, axonal transport, proteasome function and protein translation. Moreover, these three mechanisms may work separately or in synergy to cause alterations in nuclear-cytoplasmic transport, TDP-43 subcellular localization and stress granule dynamics that are toxic to motor neurons. DPR: dipeptide repeat protein; RAN: repeat associated non-ATG translation; RBP: RNA-binding protein; TDP-43: TAR DNA-binding protein 43

Before delving deeper into the role of neuroinflammation, we will first briefly outline the most important disease mechanisms at play in C9-ALS/FTD. Currently, there are three non-mutually exclusive hypotheses explaining the role of *C9orf72* HREs in disease: (1) a loss-of-function mechanism with reduced levels of *C9orf72* transcripts causing a reduction of C9orf72 protein levels, better known as C9orf72 haploinsufficiency; (2) a toxic gain-of-function mechanism with the formation of RNA foci, in which sense and antisense HRE RNAs sequester RNA-binding proteins and other proteins; (3) a toxic gain-of-function mechanism consisting of RAN translation of the sense and antisense HRE RNA resulting in toxic DPRs. These disease mechanisms are summarized in Fig. 1.

### Loss of function

The *C9orf72* gene is located on chromosome 9p21 and consists of 11 exons. While pre-mRNA transcript variants 2 and 3 encode a C9orf72-long protein (isoform A, 481 amino acids, exons 2–11), the transcript variant 1 encodes a C9orf72-short protein (isoform B, 222 amino acids, exons 2–5) [54]. In human iPSC-derived MNs the long isoform is localized to lysosomes and pre-synapses while the short isoform is localized along the nuclear membrane [55, 56]. These findings imply that they might have different cellular functions. It has been suggested that the long isoform is involved in the autophagy-lysosome pathway, while short isoform may play a role in nucleoplasmic transport [57, 58]. In fact, multiple studies conducted in a variety of (neuronal) cell lines, iPSC-derived neurons and rodent models of ALS found a link between C9orf72 haploinsufficiency and alterations in the endo-lysosomal pathway [57].

Although a reduction of some of the C9orf72 transcripts has been found in blood lymphocytes, iPSC-derived neurons, cerebellum, frontal cortex, motor cortex and spinal cord of C9-carriers [59], several other studies could not confirm this and, as a result, the C9orf72 haploinsufficiency hypothesis still remains debated in the field [60, 61]. Reduced C9orf72 protein levels have been reported in the cerebellum of C9-carriers, but to what extent C9orf72 protein levels are reduced in C9-ALS/FTD remains unclear [55]. Some of the C9orf72 antibodies used have been criticized for their lack of specificity [62]. Although reduced levels of some of the C9orf72 transcripts and reduced protein levels have been described in some studies, this could not been confirmed in other studies. The contribution of reduced C9orf72 function in ALS/FTD requires further study. As such, the role of *C9orf72* haploinsufficiency in C9-ALS/FTD remains debated. Several reviews on this topic have been recently published [57, 58, 63].

### RNA gain of function

The mechanism of RNA gain-of-function is well-established in other repeat diseases [40, 64]. The G4C2 HRE in the *C9orf72* gene can form multiple secondary structures which can bind and sequester several proteins. Both sense and antisense transcripts, containing GGGGCC or CCCCGG repeats, respectively, are being formed and identified in RNA foci [14, 65]. In most C9orf72 model systems, sense repeat RNA foci are easier to detect and more abundant than their antisense counterparts. Nevertheless, antisense foci can be robustly detected in several cell types and post-mortem samples [66].

The sense strand of the G4C2 repeat is capable of forming G-quadruplexes both at the DNA and RNA level [67–69]. G-quadruplexes are abundant in promoter and intronic regions of several genes and they are involved in multiple biological processes such as transcription, alternative splicing, translation regulation, genetic instability, telomere regulation and RNA transport and degradation [69–71]. Transcribed G4C2 RNA can bind to G4C2 repeat DNA and form R-loops, a three-stranded structure formed by a DNA/RNA hybrid, resulting in disruption of transcription and genome instability [68, 72].

Repeat-containing RNA can sequester several RNA-binding proteins and influence their function. Binding of important cellular proteins to *C9orf72* repeat RNA or their sequestration in RNA foci results in depletion of these proteins which dysfunction of multiple cellular processes. The largest group of RNA-binding protein known to bind the G4C2 repeat RNA is the heterogeneous nuclear ribonucleoprotein group (hnRNP). Sequestration of hnRNPs causes splicing alterations of several genes [40]. The role of repeat RNA toxicity is reviewed in detail elsewhere [64].

### DPR toxicity

The impact of DPRs on cellular functioning has been studied extensively. We will only briefly touch upon the different pathways that are altered by DPR expression as excellent reviews focussing on RAN translation and DPR toxicity exist [73, 74].

In general, the toxicity of the different DPRs varies, the arginine-containing DPRs, poly-GR and poly-PR, are found to be more toxic than the non arginine-containing DPRs, poly-GA, poly-PA and poly-GP [59]. The arginine-containing DPRs are capable of binding proteins that harbor low-complexity domains (LCDs) [75, 76]. As such, these arginine-containing DPRs may alter liquid–liquid phase separation, a process crucial for the formation of stress granules (SGs) [77–79]. On top of that, poly-GR and poly-PR can also affect nucleocytoplasmic transport by altering the splicing of RNA protein Ran-Gap, by binding of importins, nucleoporins (Nup) or the lamin B receptor, or by disturbing SG dynamics [80–82]. DPRs also cause nucleolar stress, leading to splicing and mRNA translation defects. Studies have demonstrated that poly-GR and poly-PR can bind several RNA-binding proteins, nuclear proteins, hnRNPs, ribosomal proteins and spliceosome components and subsequently disrupt global protein translation and RNA metabolism [78, 79, 83]. Furthermore, arginine-containing DPRs were recently found to interfere with the axonal transport machinery by binding to tubulin and motor proteins in iPSC-derived motor neurons [61, 84]. Poly-GR, as well as other DPRs, are also linked to mitochondrial and DNA damage. Feature of DNA damage and oxidative stress have been found in iPSC-derived MNs of C9-ALS/FTD patients [85]. Lastly, poly-GA, the most abundant DPR, probably acts by blocking the ubiquitin–proteasome system (UPS) as expression of poly-GA in HEK293T, neuro2a cells and primary mouse cortical neurons led to an elevated p62 expression and accumulation of ubiquitinated proteins [86].

## Major inflammatory pathways

**Fig. 2:**
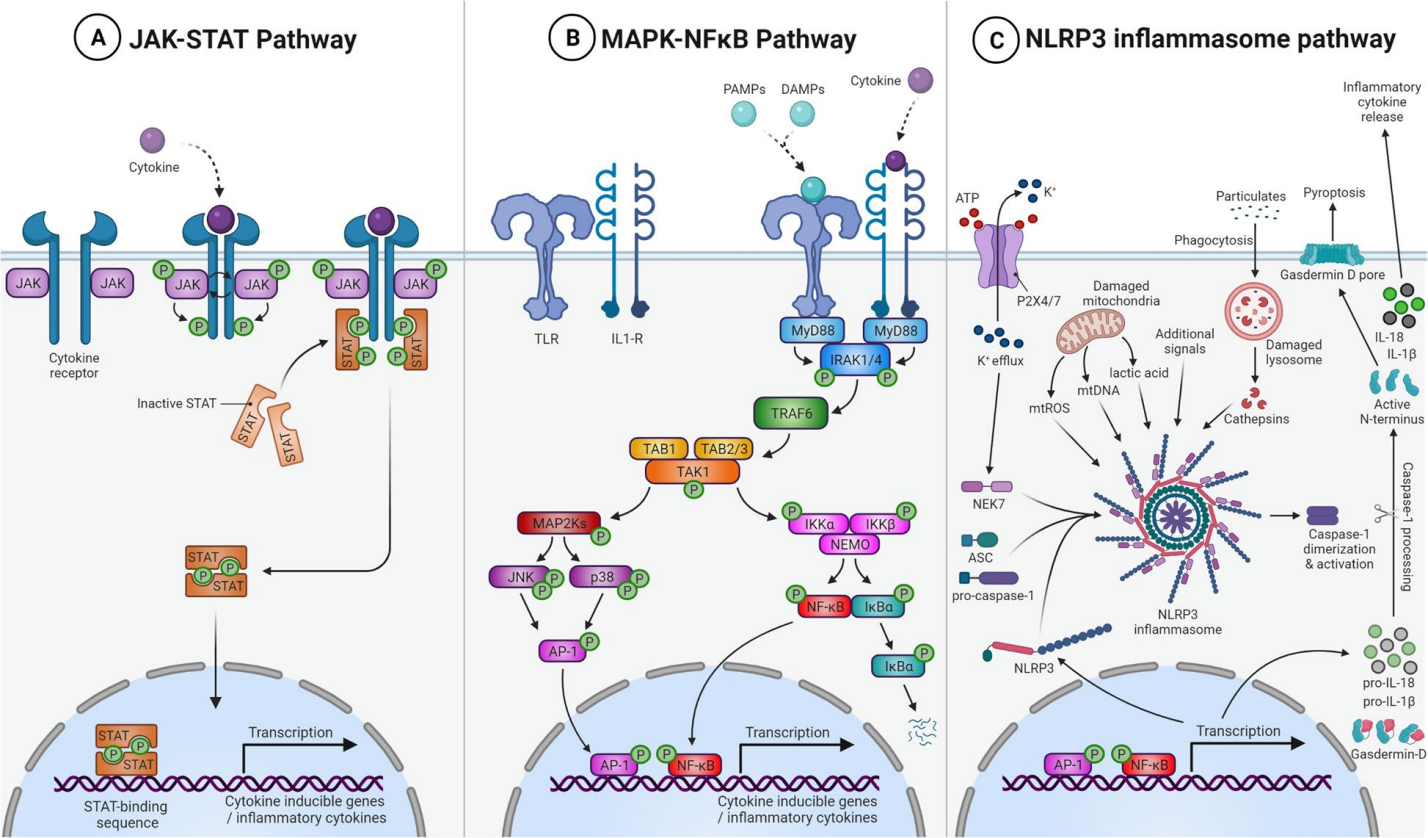
Major inflammatory pathways. **A** The JAK-STAT pathway regulates the cellular response to cytokines and growth factors and signals through dimerization of receptors upon binding of these molecules. Upon dimerization, a series of autophosphorylation and transphosphorylation events of the receptors and their associated JAK proteins results in an active STAT dimer that functions as transcription activator. **B** The MAPK and NF-κB pathway is characterized by a multi-step signaling cascade upon receptor activation eventually resulting in MAPK activation. In this cascade, generally a MAPK kinase kinase (MAP3K) phosphorylates and activates a MAPK kinase (MAP2K) which in turn activates the MAPK. Meanwhile, the MAP3K can also induce NF-κB signaling. Together with the transcription factor activator protein 1 (AP-1) activated by phosphorylation of the MAPK, NF-κB translocates to the nucleus and functions as a transcription factor. **C** The NLRP3 inflammasome pathway can be initiated by a plethora of different mechanisms that result in activation of the NF-κB pathway. The presence of additional cellular insults will result in the formation of the NLRP3 inflammasome which is able to activate caspase 1. The activated caspase 1 dimer is subsequently able to process gasdermin D, pro-IL-1β and pro-IL-18 into their mature forms causing the formation of a gasdermin D pore resulting in pyroptosis and the release of inflammatory cytokines. AP-1: activator protein 1; ATP: adenosine triphosphate; ASC: apoptosis associated speck-like protein containing a CARD; DAMP: damage-associated molecular pattern; IκBα: nuclear factor of kappa light polypeptide gene enhancer in B-cells inhibitor α; IKKα/β: Inhibitor of nuclear factor kappa-B kinase subunit alpha α/β; IL: interleukin; IL1-R: interleukin-1 receptor; IRAK1/4: interleukin-1 receptor-associated kinase 1/4; JAK: janus kinase; JNK: c-Jun N-terminal kinase; MAP2Ks: mitogen-activated protein kinase kinases; mtDNA: mitochondrial DNA; mtROS: mitochondrial reactive oxygen species; MyD88: myeloid differentiation primary response 88; NEK7: NIMA-related kinase 7; NEMO: NF-κB essential modulator; NF-κB: nuclear factor kappa-light-chain-enhancer of activated B cells; NLRP3: NLR family pyrin domain containing 3; P2X4/7: ATP-gated P2X receptor cation channel 4/7; p38: p38 mitogen-activated protein kinase; PAMP: pathogen-associated molecular pattern; STAT: signal transducer and activator of transcription; TAB1,2,3: TGF-β activated kinase 1 binding protein 1, 2 or 3; TAK1: TGF-β activated kinase 1; TLR: toll-like receptor; TRAF6: TNF receptor associated factor 6

Before reviewing the role of neuroinflammation in C9-ALS/FTD, we will shortly discuss the intracellular signaling pathways that are activated in glial cells in neuroinflammation. These three pathways are illustrated in Fig. 2.

### The Janus kinase (JAK)-signal transducer and activator of transcription (STAT)

The JAK/STAT pathway plays a critical role in the regulation of the inflammatory response. This pathway is activated by cellular responses to cytokines (including interferons and interleukins) and growth factors [87–89]. Binding of these molecules to their cell surface receptors leads to dimerization of the receptor and hence brings the receptor-associated JAK proteins in close proximity. This enables the JAKs to transphosphorylate each other, enhancing their activity. The JAKs also phosphorylate tyrosines on the intracellular domains of the cytokine receptor itself, generating binding sites for inactive STAT proteins. Upon binding, the STAT proteins themselves also get phosphorylated by the JAKs which leads to their dissociation from the receptor and subsequent dimerization/oligomerization. In this state, the STATs are translocated to the nucleus, and regulate the transcription of their target genes which comprise mostly pro-inflammatory factors. Among STATs, STAT3 may be very important in microglia and astrocytes with effects on other cell types [90–95].

### Mitogen-activated protein kinase (MAPK) and nuclear factor kappa light chain enhancer of activated B cells (NF-κB)

MAPKs are serine–threonine kinases that are involved in signal transduction of multiple extracellular stimuli including growth factors, pro-inflammatory cytokines and mitogens [96]. The MAPK pathway has been shown to be involved in several neurological conditions [97–99]. The typical and major MAPKs are characterized by multiple downstream phosphorylation events upon receptor activation ultimately leading to the activation of the MAPK. In mammals, the p38 kinases, c-JUN N-terminal kinases (JNKs) and extracellular signal-regulated kinases (ERKs) are the three major MAPK families. While the ERK family of MAPKs is responsible for differentiation and proliferation signalling upon growth factor and mitogen binding, the JNKs and p38 kinases (highly expressed in glial and neuronal cells) are stress-activated protein kinases primarily activated through various environmental stresses and cytokines [100]. Upon binding of the signalling molecules to various receptors, a multi-step signalling cascade is initiated. In many cases, myeloid differentiation primary response protein 88 (MyD88) recruits interleukin-1 receptor-associated kinase-4 (IRAK4). This complex will subsequently recruit, phosphorylate and activate other IRAKs (e.g. IRAK1 and IRAK2). Activated IRAKs are able to activate TNF receptor-associated factor 6 (TRAF6) which in turn activates the complex composed of transforming growth factor-β-activated kinase 1 (TAK1) (a MAPK kinase kinase or MAP3K) and TGF-beta activated kinase 1 binding protein 2 and 3 (TAB2/3). This complex can then activate MAPK kinases (MAP2Ks), but also induce the NF-κB signalling pathway. On its turn, MAP2K phosphorylates the p38 or c-Jun N-terminal kinase (JNK) MAPKs, which results in the production of the transcription factor activator protein 1 (AP-1). Finally, the NF-κB and activator protein 1 (AP-1) transcription factors translocate to the nucleus and activate the transcription of cytokine inducible genes.

### NOD-like receptor family pyrin domain containing 3 (NLRP3) inflammasome

The first step in NLRP3-mediated inflammation is triggered by the binding of pathogen-associated molecular pattern molecules (PAMPs) or damage-associated molecular pattern molecules (DAMPs) to Toll-like receptors (TLRs) or nucleotide-binding oligomerization domain and leucine-rich repeat-containing receptors (NLRs), or by the binding of endogenous cytokines to their respective receptors ultimately activating the transcription factor of NF-κB signalling pathway. This, in turn, results in the increased transcription of the NLRP3, pro-IL-1β and pro-IL-18, two pro-inflammatory cytokines. The second step in the NLRP3 inflammasome activation cascade requires some kind of cellular insult such as mitochondrial damage, reactive oxygen species (ROS) generation, lysosomal dysfunction or ionic flux dysregulation. Ultimately, this will result in the interaction of NLRP3 and apoptosis associated speck-like protein (ASC), inducing caspase 1 activation. This leads to cleavage, maturation and eventually the secretion of IL-1β and IL-18, mediating inflammation and the innate immune response [101, 102]. In addition, active caspase 1 also cleaves the protein gasdermin D, which enables it to oligomerize and form a gasdermin D membrane pore. This causes pyroptosis, an inflammatory mediated cell death that is triggered by proinflammatory molecules and occurs as the result of membranous pore formation, cytoplasmic swelling, and leakage of cytosolic contents [103, 104].

## Neuroinflammation in C9-ALS/FTD

**Fig. 3:**
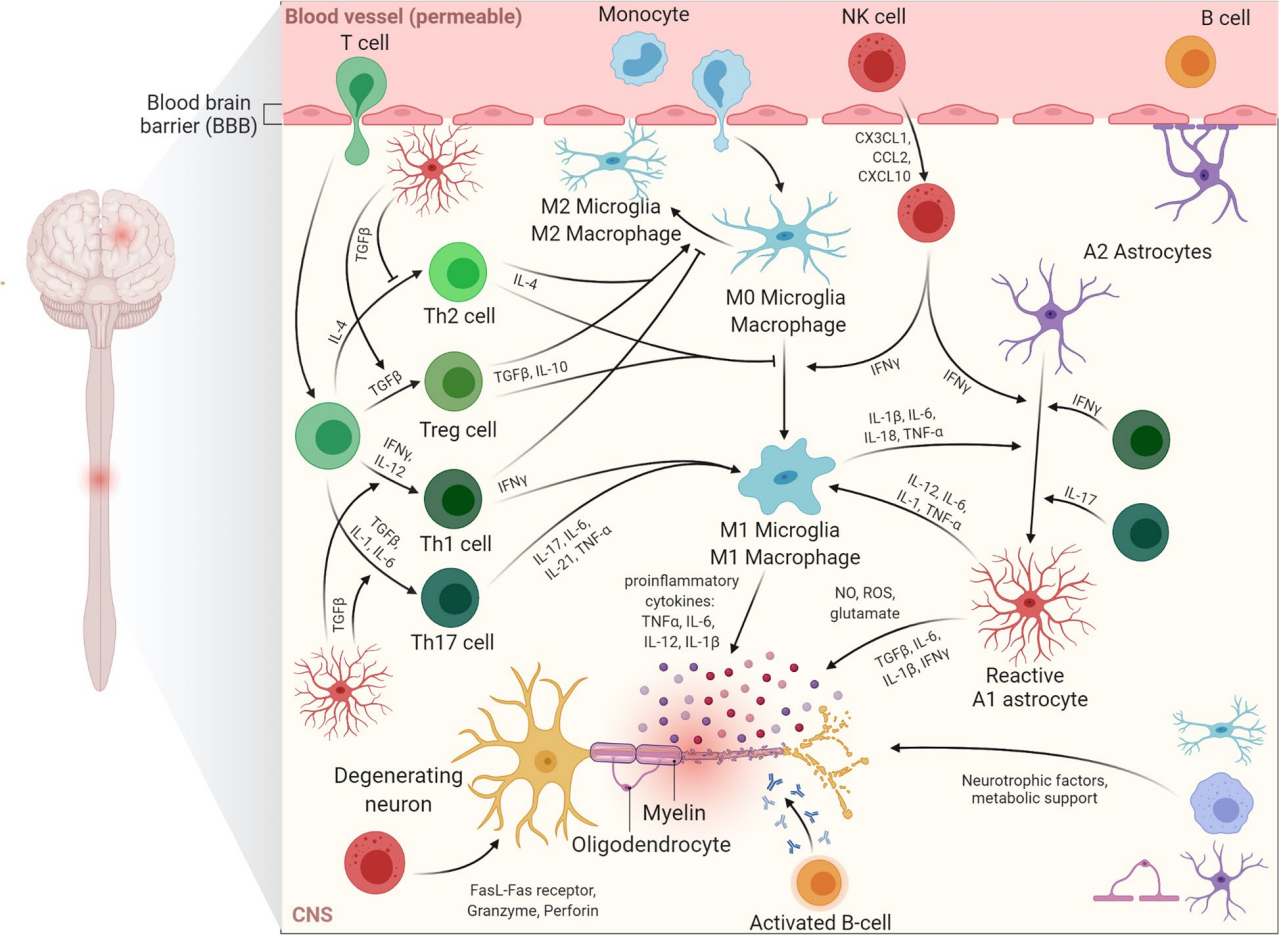
Overview of the neuroinflammatory pathways at play in C9-ALS/FTD. Coinciding with the neurodegeneration, astrocytes and microglia undergo a switch towards a neurotoxic and (over)activated state in which they produce and secrete an array of pro-inflammatory factors including cytokines (TNFα, IL-6, IL-12, IL-1β, TGFβ, IFNγ), glutamate, NO and ROS. During disease progression, peripheral immune cells such as T cells, B cells, monocytes and NK cells migrate into the CNS. Upon CNS infiltration, monocytes may differentiate to macrophages that act similar to the CNS-residing microglia. Although an active involvement of T cells in ALS pathophysiology is debated, disease progression has been associated with a disbalance between neuroprotective and neurotoxic T cell populations. Next, NK cells are attracted to the CNS by chemokines and can stimulate neurotoxic glia differentiation or directly be toxic to the neurons. Finally, although their role has never been directly demonstrated, activated B cells may exert toxicity by the secretion of autoimmune antibodies. CCL2: C–C motif chemokine ligand 2; CNS, central nervous system; CX3CL1: CX3 chemokine ligand 1; CXCL10: C-X-C motif ligand 10; IFNγ, interferon γ; IL, interleukin; NK cell: natural killer cell; NO, nitric oxide; ROS, reactive oxygen species; TGFβ, transforming growth factor β; TNFα, tumor necrosis factor α

Even though the exact role of neuroinflammation in neurodegeneration still remains debated, dysregulation of the immune system is a pathological hallmark in nearly all neurodegenerative diseases [105–107]. It consists of the activation of glial cells with an increased activation of the proinflammatory MAPK and NF-κB signalling pathways, increased cell surface expression of cell adhesion molecules, increased cytosolic expression of proinflammatory enzymes and an increased release of proinflammatory secretory proteins such as cytokines, chemokines and growth factors [108–112]. While neuroinflammation is mainly studied in patients and in vivo models systems due to the inherent nature of the process that requires multiple cell types and secreted molecules to interact, recent advances in the field of iPSCs made it also possible to investigate some deregulated processes in relevant cell types in vitro. In addition, iPSCs also enable the study of cell autonomous mechanisms of deregulated processes as cells are naïve to interactions with other cell types. This is summarised in Fig. 3.

### Glial cells

Glial cells or neuroglia are non-neuronal cells located in the central nervous system (CNS) and are responsible for CNS homeostasis, structural and trophic support for neurons, removal of pathogens and modulating neurotransmission. While neuroglia include multiple cell types such as oligodendrocytes, satellite cells and Schwann cells, we will focus on microglia and astrocytes in this review as these cell types have been shown to have a major role in the regulation of neuroinflammation.

#### Microglia

Microglia are the resident innate immune cells of the CNS, making up 5–15% of all cells in the brain [113, 114]. As such, they are the first responders to pathological injuries and have a crucial role in the maintenance of homeostasis in the CNS [106, 115–118]. Microglia are able to interact with both immune cells residing in the CNS as well as with peripheral immune cells that infiltrate into the CNS resulting in multiple crosstalk events [119, 120]. Microglia exist in various forms depending on their activation status. In the early stages of neurodegenerative diseases, anti-inflammatory cytokines such as transforming growth factor beta (TGF-β), glial cell-derived neurotrophic factor (GDNF) and some interleukines (IL-4, IL-10 and IL-13) guide microglia to a neuroprotective state (M2 microglia) leading to the release of diverse neuroprotective factors such as insulin-like growth factor (IGF-1) [121, 122]. Furthermore, regulatory T-cells (Tregs) and the neuroprotective M2 microglia downregulate the proliferation and toxic function of Th1 cells and toxic microglial cells (M1 microglia). However, in a later stages of disease, glia and immune cells transform towards an opposite and more cytotoxic phenotype. This response is a result of the secretion of ROS, pro-inflammatory cytokines such as IL-1β and IL-6, tumor necrosis factor-alpha (TNF-α) and chemokines including CCL2 (C–C motif chemokine ligand 2) [121, 123]. Ultimately, this leads to the suppression of Tregs and an increased production of interferon-gamma (IFN-γ) by T helper 1 (Th1) cells [124, 125]. Taken together, upon sustained neuroinflammation, the neuroprotective microglial response switches from a protective to a neurotoxic response [116]. Eventually, the inability for neuronal repair and the dystrophic morphology of microglia will lead to synaptic impairment, oxidative damage and mitochondrial dysfunction, which in turn will result in aggravation of neurodegeneration [121, 126–128]. The concept of M1 and M2 microglia is an oversimplification which does not fully reflect the inflammatory cell states of microglia in neurodegenerative diseases. The recent advent of novel technologies, such as single-cell RNA sequencing, mass cytometry and proteomics, has facilitated the profiling of single cells with high-throughput technologies [129, 130]. There is increasing evidence for subclasses of microglial cell states that are seen in neurodegenerative diseases [131]. However, the heterogeneity of microglia across different CNS regions and their functionality during disease needs to be further investigated.

Cellular studies that aimed to decipher the role of C9orf72 and the consequences of its haploinsufficiency in microglia mostly pointed towards an involvement of C9orf72 in the endo-lysosomal pathway [57]. Unfortunately, the differentiation protocols for iPSC-derived microglial cells have only recently emerged and hence only limited in vitro data concerning the role of C9orf72 in microglia l cells is available [132]. One study has used C9-ALS/FTD iPSC-derived microglia and showed that microglia produce DPRs and have reduced C9orf72 protein levels [133]. Although transcriptional profiles between *C9orf72* microglia and control cells were found to be highly similar with only 27 genes being differentially expressed, they did observe a dysfunction of the endo-lysosomal pathway. This was supported by changes in lysosomal associated membrane protein 1 (Lamp1) and early endosome antigen 1 (EEA1) levels, which was accompanied by defects in phagocytosis [133]. On top of that, stimulation of the inflammatory phenotype in these microglia with lipopolysaccharides (LPS) and amyloid beta (Aβ) resulted in an augmented inflammatory response [133]. Interestingly, these in vitro phenotypes closely resemble those found in microglia of postmortem tissues from C9-ALS/FTD patients [134, 135]. Another recent study explored the possible dysfunction and therapeutic potential of *C9orf72* patient iPSC-derived M2 macrophages. Surprisingly, the presence of the *C9orf72* HRE did not affect the immunomodulatory activity of the M2 microglial cells and still allowed them to convert blood-derived Teffs into functional Tregs and counteract the activation of M1 microglia [136].

As C9orf72 is highly expressed in myeloid cells (including microglia), the elucidation of its role in microglia l function has received a lot of attention in several knockout *C9orf72* mouse models. Progressive splenomegaly and lymphadenopathy, increased levels of pro-inflammatory cytokines, autoinflammation with the production of autoantibodies, and auto-immune like diseases have all been reported in both *C9orf72* + */ − *and *C9orf72 − / − *knockout mouse models [134, 137–139]. These findings suggest that *C9orf72* haploinsufficiency is sufficient to drive an altered myeloid cell function and systemic immune response [134, 137]. Interestingly, these knockout mouse models did not display a neurodegeneration phenotype consistent with ALS/FTD, indicating that the deletion of *C9orf72* alone is not sufficient to cause C9 ALS/FTD. Nevertheless, microglia-induced MN death in ALS is in part caused by downregulation of NF-kB signaling in microglia and attenuating the proinflammatory microglial activation can prolong survival in ALS mice [139–141]. In this last study, Zhao and colleagues found evidence for activation of the NLRP3 inflammasome pathway by TDP-43 selectively in microglia as motor neurons did not exhibit neurotoxic effects [141]. Of note, this pathway can be activated by numerous other pathological mechanisms at play in C9-ALS/FTD, such as mitochondrial damage, lysosomal dysfunction and the presence of protein aggregates. In both C9orf72 − / − macrophages and C9orf72 − / − microglia, lysosomal accumulations and increased production of circulating proinflammatory cytokines, such as IL-6 and IL-1β, as a result of hyperactive immune responses, have been reported [34]. Moreover, the spinal cord from *C9orf72* -/- mice, poly-PR (GFP-PR28) heterozygous overexpression mice and Thy1 (GA)149-CFP overexpression mice all showed an upregulation of inflammatory pathways [142–144].

As we have discussed in detail in a recent review, the C9orf72-SMCR8-WDR41 complex is an initiator of autophagy and lysosomal function [57]. *Smcr 8-/-* mice have similar inflammatory phenotypes as *C9orf72-/-* mice consisting of splenomegaly, lymphadenopathy, activated circulating T cells and excessive inflammatory cytokine production [145]. TLRs play crucial roles in the innate immune system mediated through NF-κB and interferon regulatory factors (IRFs) signalling which result in the production of inflammatory cytokines or chemokines. In a *Smcr8 *^*−/−*^ mice model, the autoimmunity and inflammatory phenotypes that closely resemble the ones found in C9-ALS/FTD were rescued by triple knockout of the endosomal TLRs [146].

In an adeno-associated virus (AAV) gain-of-function C9-ALS/FTD mouse model, accumulation of RNA foci and DPR proteins, TDP-43 pathology, gliosis, neurodegeneration and behavioral abnormalities have been reported [147, 148]. In poly-GA and poly-PR mouse models, the role of inflammation and TDP-43 abnormalities in neurodegeneration have been investigated [149]. By performing bulk RNA sequencing of brainstem, cortex, hippocampus and spinal cord, it was shown that poly-GA mouse models have a strong immune response including upregulation of interferon-inducible genes. At the end stage of disease, activation of an interferon pro-inflammatory microglial signature has been reported in poly-GA mice compared to poly-PR mice. These findings were also confirmed in the spinal cord of C9-ALS patients [149].

#### Astrocytes

Astrocytes make up another dominant cell population in the brain. Astrocytes play a significant role in the maintenance of the blood brain barrier (BBB), they provide metabolic support to neurons, aid in axonal health maintenance, protect against oxidative stress, restrict the spread of cytotoxic inflammation and regulate the neuroendothelial permeability [150–153]. Similar to microglia, astrocytes can switch from a protective to a neurotoxic activated phenotype, commonly referred to as A2 and A1 astrocytes respectively. Activated astrocytes produce and release multiple proinflammatory molecules such as cytokines, chemokines and small molecules including ROS and nitric oxide (NO), all of which are toxic to MNs. In addition, these factors can activate microglia and recruit infiltrating peripheral immune cells to the CNS further exacerbating the neurodegenerative process by suppressing neuronal supporting mechanisms [119, 154–157]. Like microglia, astrocyte heterogeneity across the CNS regions and age-dependent changes in gene expression that may contribute to neuroinflammation have been reported [158]. Nevertheless, the functional implications of these changes in gene expression and cellular motor neuron-immune cell crosstalk are still lacking.

iPSC-derived astrocyte models of C9-ALS/FTD have been around for several years now, and co-culture experiments with MNs have revealed that the toxic effects of astrocytes on motor neurons are mediated through physical contact as well as by soluble factors [159–161]. In one study, however, replacement of the cell culture medium by control astrocyte conditioned medium (ACM) was insufficient to reverse the MN cell death [160]. Moreover, impairment of autophagy, increased SOD1 levels and activation of inflammatory pathways, especially the NF-κB signalling pathway, might play a role in this toxicity [135, 159, 162]. Interestingly, knockdown of *C9orf72* in astrocytes proved to have profound effects on astrocyte-mediated neurotoxicity as this resulted in p62 inclusion formation and major dysfunctions in glutamate homeostasis (e.g. intracellular glutamate accumulation and impaired glutamate uptake by decreased levels of the astrocytic excitatory amino acid transporter 1 and 2 (EAAT1-2)) [163]. Additional investigations led to the finding that both NFκB and the shorter C9orf72 isoform can bind to the endothelin 1 (EDN1) promotor site, a negative regulator of EAAT2 [163]. In addition, iPSC-derived astrocytes harboring *SOD1* or *C9orf72* mutations, and astrocytes derived from sALS patients, did exert neurotoxic effects on MNs and presented with disease-associated astrocytic phenotypes [164–167]. More specifically, these neurotoxic effects were mediated by a decrease in the magnitude of voltage‐activated Na^+^ and K^+^ currents, a decrease in the secretion of antioxidants and an increase in astrocytic oxidative stress and premature cell senescence [165]. Finally, in vivo transplantation of human astrocyte progenitor cells in the mouse spinal cord revealed that astrocytes from sporadic ALS patients induced a neuroinflammatory phenotype in these mice accompanied by ubiquitin aggregates, movement decline and a reduction in MN numbers [168].

Although several studies reported on the toxicit of C9-ALS/FTD astrocytes, comparison between these studies remains challenging due to differences in the methods used, such as the astrocyte differentiation protocols. For instance, one study generated iPSCs from fibroblasts before differentiating them into astrocytes in chemically defined media (31,841,614), while another study first generated neural precursors from adult skin fibroblasts before differentiating them into astrocytes using serum-containing media (24,379,375). As a consequence of these methodological differences, different transcriptional patterns with changes in functionality may be present in those astrocytes ( 27,725,795). Differences in the purity of cultures andthe use of primary cultures of mutant *C9orf72* expressing mouse astrocytes may explain differences in study results (31,841,614). Future studies are needed to investigate the effect of mutant C9orf72 astrocytes on MNs survival. In addition, the exact role of astrocyte-mediated toxic factors (e.g. ROS, proinflammatory cytokines and effectors of necroptosis and apoptosis) and the astrocyte secretome as mediators of MN homeostasis needs to be further investigated.

#### Motor neurons

Although neurons are not key players in the actual inflammation process, alterations in their cellular homeostasis can make them more or less vulnerable to inflammatory stimuli and hence contribute to inflammation-induced MN loss. One such example was recently identified in human induced motor neurons (iMNs) carrying the *C9orf72* HRE. In these cells, the reduction of C9orf72 protein levels led to an increase of glutamate receptors on the iMNs membrane causing excitotoxicity in response to extracellular glutamate [63]. As mentioned above, excitotoxicity can further be enhanced by reduced glutamate clearance by astrocytes by loss of EAAT2 glutamate transporters [169, 170]. On top of that, neuronal damage itself may cause the release of self-antigens and proinflammatory factors that activate resting astrocytes and microglia. These will, as mentioned before, produce neurotoxic factors such as NO, ROS, pro-inflammatory cytokines resulting in a viscous feedback-loop that aggravates the neuroinflammation in the CNS [141, 171].

### Peripheral immune system

Under normal conditions, the CNS immune system and peripheral immune cells are separated by the BBB and blood-cerebrospinal fluid barrier (B-CSF-B) which limit the entry of circulating toxic molecules and pathogens into the CNS [171]. In case of injury, the peripheral immune system will invade the CNS causing neuroinflammation by infiltration of leukocytes and macrophages [172–174].

#### Monocytes/Macrophages

Monocytes are a central constituent of the peripheral innate immune system which are derived from precursors in the bone marrow. Monocytes can differentiate further into macrophages or dendritic cells (DCs) as they migrate into tissues and participate in the response to an inflammatory stimulus. Macrophages have multiple functional similarities with microglia in the CNS. First, macrophages express TLRs and therefore can be activated by PAMPs, endogenous molecules released by injured tissues and necrotic cells, such as DAMPs and aggregated proteins [175]. Second, they have the capability to activate Th1 cells by presenting antigens through expression of major histocompatibility complex (MHC) class II molecules [176]. Third, by interacting with Th cells and Tregs they can differentiate into either an anti-inflammatory and neuroprotective M2 phenotype or pro-inflammatory and neurotoxic M1 phenotype [177].

Toll-like receptor 4 (TLR4), a transmembrane protein that is involved the recognition of PAMPs and DAMPs is upregulated in peripheral blood mononuclear cells obtained from sALS patients, suggesting a chronic activation of monocytes in ALS [178]. In addition, there are several reports of increased CNS infiltration of monocytes in ALS patients, and of altered CNS infiltration in asymp-C9, both suggesting the possibility that CNS invading monocytes may have a modifying role in the context of ALS pathogenesis [179, 180]. Moreover, elevated levels of proinflammatory molecules including CCL2, IL-1b, IL-8, FosB, C-X-C motif ligand (CXCL)-1 and CXCL2 have been documented in the cerebrospinal fluid (CSF) and spinal cord of ALS patients [109, 179, 181, 182]. There is also evidence for an imbalance between M1 and M2 macrophages in ALS pathophysiology [183, 184]. M1 macrophages in ALS patients have been demonstrated to produce more pro-inflammatory cytokines than M1 macrophages from healthy individuals [185]. Notably, among these pro-inflammatory cytokines, IL-6 and TNFα levels are positively correlated with disease burden and disease progression, respectively. A positive correlation between the disease progression rate and macrophage infiltration and complement secretion in peripheral nerves has also been reported [186–188].

Among the myeloid cells, C9orf72 is highly expressed specifically in CD14 + monocytes [189, 190]. Interestingly, Rizuu et. al, showed the presence of RNA foci in CD14 + monocytes in brain tissue of C9-carriers [190]. In *C9orf72* -/- mice, the levels of IL-12, IL-17a, IL-10 and TNF in serum were increased compared to wild-type mice [137]. Moreover, RNA sequencing studies from brain and lymphoid tissues revealed perturbations in genes involved in immune responses.

#### T cells

T cells, originating from lymphoid stem cells in bone marrow progenitors and maturing in the thymus, express T cell receptors (TCR). On their surface, T cells can express either CD8 glycoprotein (cytotoxic CD8 + T cell) or CD4 glycoprotein (CD4 + cells). Furthermore, CD4 + naive cells differentiate into different subsets called T helpers (Th1, 2, 9, 17, 22), Treg, and follicular helper T cells (Tfh). These subset are characterized by different cytokine profiles and are involved in the elimination of intracellular pathogens and in the attenuation of neuroinflammatory processes [191–193]. In many neurodegenerative diseases CD4 + T cells infiltrate the CNS where they interact predominantly with microglial cells and promote neuroinflammation ultimately leading to neuronal damage and neurodegeneration [127, 194]. Infiltration of T cells and hyperactivation of resident immune cells has been demonstrated in post-mortem spinal cords of ALS patients [109, 195]. CD4 + cells are detected near the ventral horn and corticospinal tract, implicating their infiltration into the CNS during the disease course [120]. Tregs are a key cell type responsible for suppressing the immune response, thereby maintaining homeostasis and self-tolerance [196]. Tregs have shown to be neuroprotective in ALS and levels of Tregs have been shown to inversely correlate with disease progression rates [183, 197, 198]. CD8 + cells infiltrate in the CNS and have the capability to participate in regulation of the immune response by producing cytokines such as IFN-γ and TNF-α [199, 200]. Unlike CD4 + cells that are involved in different stages of the disease, the CD8 + cells have only been observed in later stages of the disease [198, 200–202]. CD8 + cells positively correlate with neuronal function in the peripheral nerves [203]. Increased levels of CD4 + cells, decreased levels of CD8 + cells and Tregs (CD4 + /CD25 +) in peripheral blood were detected in ALS patients compared to healthy individuals [204]. However, to what extent peripheral T cell contribute to ALS pathogenesis is controversial and the question remains whether the level of circulating T cells is of importance to disease-related immune responses in ALS. Increased levels of CD4 + cells and decreased levels of CD8 + cells and Tregs (CD4 + /CD25 +) were detected in ALS patients compared to healthy individuals [204]. Little is known about T cells in C-ALS/FTD patients. Studies in *C9orf72* -/- mice revealed some interesting signs of immune system dysregulation [137], such as: 1) significantly increased percentages of CD8 + cells expressing programmed death protein 1, 2) increased CD69 and CD44 (markers of effector memory T cells) levels in spleen and kidney, 3) increased CD44, CD69 and PD-1 expression in CD4 + cells in spleen, kidney and cervical lymph node, 4) increased percentages of Tregs in spleens and cervical lymph nodes, and 5) reduced expression of CD62L and CD127 (markers of the naïve and central memory T cells) in spleen. These kind of studies in C9-ALS/FTD patients are needed to explore the role of T cells and their subpopulations in neurodegeneration in patients.

#### NK cells

Natural killer cells (NK) are bone marrow-derived hematopoietic cells that are capable of directly attacking and killing target cells through interaction of FasL protein and the Fas receptor which triggers apoptosis through the classical caspase cascade and by the release of cytotoxic granules containing granzyme and perforin [205, 206]. As such, NK and CD8 + T cells are both cytotoxic effector cells of the immune system. Additionally, NK cells are able to produce chemokines and cytokines through stimulation of activatory or inhibitory receptors.

In case of CNS inflammation, peripheral NK cells are recruited to the CNS by chemokines such as CX3C ligand 1 (CX3CL1), CCL2 and CXC ligand 10 (CXCL10), produced by neurons and microglia, astrocytes, and other inflammatory cells, respectively [207, 208].

Although there is some evidence for a role of NK cells in ALS, little is known in the context of C9-ALS/FTD. In the spinal cord of end-stage mSOD1 mice, the presence of NK cells has been demonstrated [201]. In the blood of ALS patients and in post mortem ALS spinal cord and motor cortex, an increased number of NK cells has also been reported [209]. In addition, a CCL2-dependent accumulation of NK cells in the motor cortex and spinal cord of hSOD1^G93A^ and TDP43^A315T^ ALS mice models has been described [210]. These NK cells mediate microglia and astrocyte proinflammatory polarization via secretion of IFNγ and inhibit CNS infiltration of Treg cells. Moreover, since MNs express natural killer group 2D (NK2GD) ligands, NK cells may also directly induce MN death [210]. Interestingly, depletion of NK cells slows down the rate of neurodegeneration and increases the survival in these mice models of ALS, further underscoring the involvement of NK cells in both the onset and progression of MN death in ALS [210]. While the involvement of NK cells in the modulation of neuroinflammation in neurodegenerative disease has been reported [211–213], the precise role of NK cells as protective or deleterious factors in ALS and FTD specifically needs to be further explored.

A recent study showed changes in lymphocyte subpopulations including NK cells, CD4 + naïve cells and CD8 + cells subtype, effector memory cells, in C9-ALS patients compared to non-C9-ALS patients [214]. NK and T cells can presumably act as prognostic markers for survival. However, a large cohort study is required for a more in-depth validation.

## A special focus on STING

**Fig. 4:**
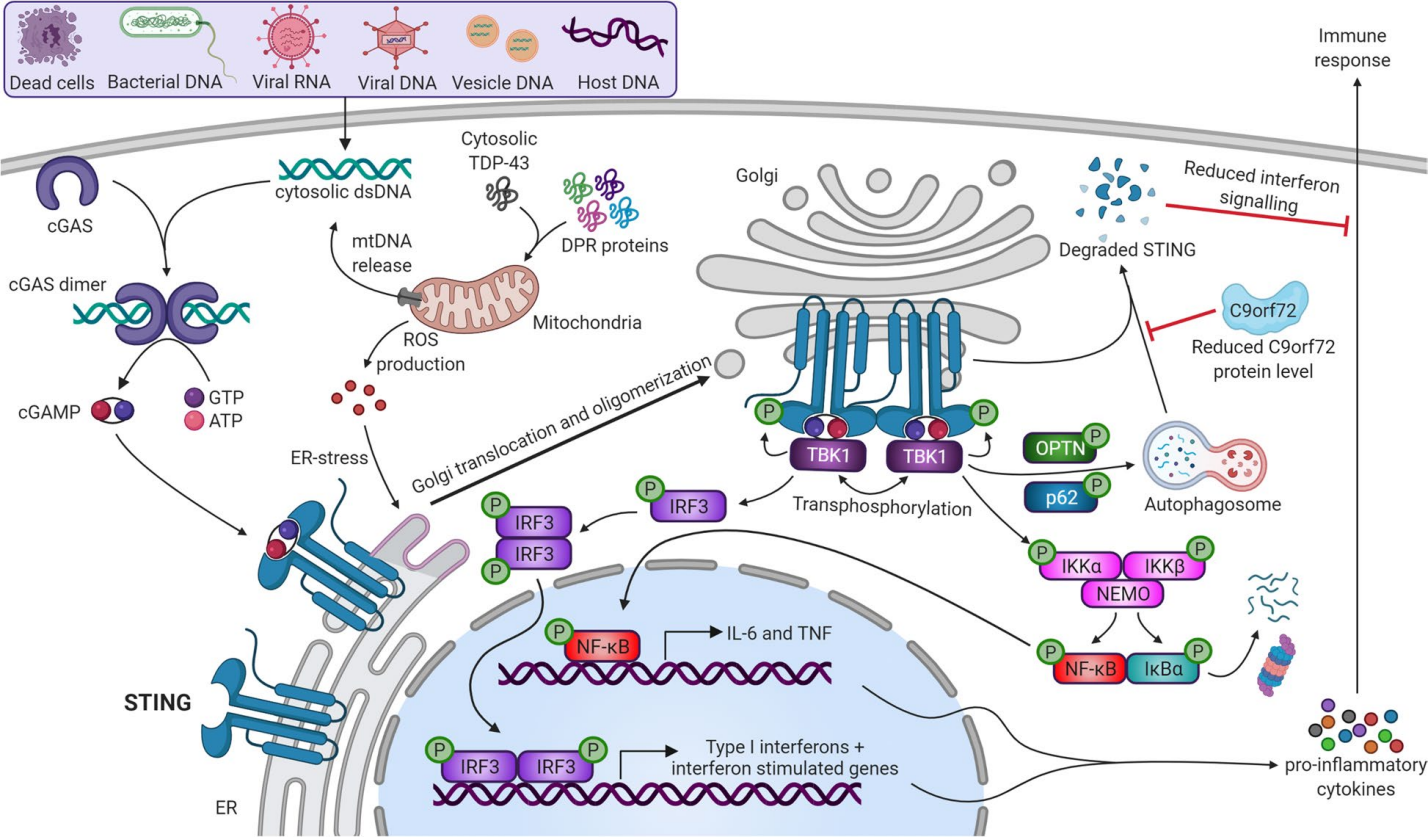
Overview of the STING pathway, the involvement of C9orf72 and its relation to C9 ALS/FTD. Cytosolic (mislocalized) TDP-43 species and/or DPR proteins cause mitochondrial damage and lead to the increased production and release of toxic ROS and mitochondrial DNA (mtDNA). cGAS, which is a cytosolic DNA sensor, binds the mtDNA and catalyzes the production of cGAMP which in turn results in stimulator of interferon genes (STING) protein dimerization. STING dimers are able to translocate to the Golgi, where they bind and activate TANK binding kinase 1 (TBK1). This complex will then phosphorylate inhibitor of NF-κBα (IκBα) and interferon regulatory factor 3 (IRF3) and cause both transcription factors to localize to the nucleus and promote the transcription of proinflammatory cytokines and type I interferons. The active STING-TBK1 signalosome is involved in autophagosome formation, an initial step in the process of autophagy. Interestingly, STING is degraded itself by autophagy and thus has an elegant self-regulatory mechanism to control its levels and the activation of the STING-based immune response. Moreover, as C9orf72 is also involved in endolysosomal trafficking and autophagy, reduced levels of the C9orf72 protein may cause a delay or failure of STING degradation and a hyperactivation of the type I interferon response. ATP: adenosine triphosphate; cGAS: cyclic guanosine monophosphate–adenosine monophosphate (cGAMP) synthase; GTP: guanosine triphosphate; IL-6: interleukin 6; IRF3: interferon regulatory factor 3; mtDNA: mitochondrial DNA; NFκB: nuclear factor-κB**;** OPTN: optineurin; P: phosphorylation; P62: sequestome 1/ ubiquitin-binding protein p62; STING: stimulator of interferon genes; TBK1: TANK binding kinase 1; TDP-43: TAR DNA-binding protein 43; TNF: tumor necrosis factor

Recently, a new key player in the field of immunity, has been linked to ALS and C9-ALS/FTD [215, 216]. This pathway is called cyclic GMP–AMP synthase (cGAS)–stimulator of interferon genes (STING) pathway as illustrated in Fig. 4. Besides being a crucial component of the innate immune system, STING signaling is also involved in autophagy, apoptosis and necroptosis [217–219]. Cytosolic DNA is sensed by a DNA sensor protein cGAS to catalyze cyclic GMP–AMP (cGAMP) by using ATP and GTP as substrates. This generates the second messenger cGAMP which then binds to the ER-localized adaptor protein STING. Translocation from the ER to the Golgi and subsequent phosphorylation occurs. The phosphorylation can also occur through ER-stress by ROS production as a result of cytosolic TDP-43 and DPR protein in the mitochondria. Additionally, TBK1 phosphorylates IRF3, leading to dimerization, nuclear translocation and eventually activation of the transcription of genes encoding type I interferons such as interferon-β (IFNβ). IκB kinase (IKK) phosphorylates NF-κB inhibitor IκBα, resulting in nuclear translocation of NF-κB. This will activate the transcription of genes encoding for proinflammatory cytokines such as IL-6 and TNF and eventually trigger the immune response (Fig. 4). Recently, a link between TDP-43 and the STING pathway has been elucidated [216, 220]. In iPSC-derived MNs from TDP-43 ALS patients and mutant TDP-43 mice, TDP-43 was found to mislocalize to the cytoplasm and enter the mitochondria. This mitochondrial localization of TDP-43 induced ROS production and the release of mitochondrial DNA, which activated the STING pathway [220].

Interestingly, a study has reported an early upregulation of the type I interferon response and adaptive immune activation in DCs isolated from *C9orf72* -/- mouse models, suggesting involvement of the STING-induced inflammation in C9orf72 pathology [221–223]. The STING signalling pathway is mainly regulated by degradation of the STING protein through autophagy [138]. As the C9orf72 protein is involved in endolysosomal trafficking and autophagy, a loss of C9orf72 levels results in the delayed degradation of the STING protein and an increased activation of the type I interferon response. Moreover, pharmacological inhibition of this pathway by the use of a STING antagonist was able to mitigate the hyperactive type I interferon response, further underscoring the STING pathway as potential therapeutic target [222].

## The possible role of the gut microbiome

There is accumulating evidence that gut microbiomes contribute to neurodegenerative diseases [224–226]. Differences in gut microbiota have been reported between ALS cases and controls [226]. Nevertheless, some studies found evidence for changes in the gut microbiome, but this could not be confirmed by other studies [227–233]. Wether these changes are related to dietary changes or truly contributing to neurodegeneration requires further study.

Recently, the role of gut microbiota has been investigated in *C9orf72* knockout mouse models [234]. This was studied in 2 colonies including *C9orf72Harvard* and *C9orf72Broad mice*. Compared to *C9orf72Broad* mice*, C9orf72Harvard* mice had a shorter lifespan, severe movement problems and elevated blood inflammatory markers, such as elevated cytokines and autoimmune antibodies. In the microbiome of *C9orf72Harvard* mice, murine norovirus, *Helicobacter*, *Pasteurella pneumotropica* and *Tritrichomonas muris* were more commonly detected and a reduced microbiome was found, in comparison with *C9orf72Broad*. Interestingly, treating the *C9orf72Harvard* with the transplants of faecal microbiota from *C9orf72Broad* mice. Treatment with antibiotics could revert this inflammatory phenotype and reduce the infiltration of neutrophils and CD3e + T cells in the spinal cord, highlighting a possible role of gut microbiota-mediated inflammation in the CNS.

## Evidence in C9-ALS/FTD patients

### Blood studies

A systemic immune response in patients with neurodegenerative brain diseases may, first of all, be measured through elevation of inflammatory cytokines, immune cells and other markers of inflammation in the blood.

Clinical studies comparing blood-based markers of inflammation in C9-ALS/FTD patients are scarce. One group found no differences in plasma MCP-1, ‘regulated upon activation, normal T cell expressed and presumably secreted’ (RANTES), IL-8, or IL-1β, but reported a trend towards increased IL-10 levels in C9-carriers [235]. Another study reported a significantly decreased level of high density lipoprotein (HDL) in FTD patients with a *C9orf72* HRE versus FTD patients without such mutation [235]. A decrease in HDL is a marker of systemic inflammation [236]. The levels of very low- and low-density lipoprotein subclasses, high sensitivity C-reactive protein (hs-CRP) or glycoprotein acetyls (GlycA) were not different. A recent study of biomarkers of inflammation in ALS patients and healthy controls revealed an increased plasma TNF‐α level in C9-ALS patients in comparison with non-C9-ALS patients and controls, suggesting a pronounced role of inflammation in C9-ALS [237]. Less is known about changes of blood inflammatory markers in FTD. Some studies reported signs of systemic inflammation in FTD patients, such as increased levels of IL-6 [238].

### CSF studies

Several studies have reported changes of inflammatory biomarkers in the CSF from patients with FTD and ALS.

In C9-ALS/FTD patients, relatively few studies of neuroinflammatory CSF biomarkers have been performed. A recent comparison between low numbers of FTD patients with different mutations did no identify increased CSF levels of chitotriosidase (CHIT1) and YKL-40 in *C9orf72* HRE patients [239].

A study comparing CSF biomarker profiles in ALS patients with slow and fast disease progression and controls pointed towards more neuroinflammation in C9-ALS [240]. While CSF IL‐15 and soluble triggering receptor expressed on myeloid cells 2 (sTREM2) levels were not elevated in the ALS cohort as a whole, they were elevated in the fast‐progression and the C9-ALS patient groups. In addition, MCP-1 and IL-18 levels were more elevated in CSF from the C9-ALS and the fast‐progressing patients. TNF-ɑ was only elevated in the C9-ALS patients [237]. Another recent study confirmed a prominent inflammatory CSF signature in C9-ALS patients [241]. However, as most C9-ALS patients have a fast disease progression, these changes may be associated with a fast disease progression rather then with the C9 genotype as such.

Another study looked at presymptomatic and symptomatic carriers of *C9orf72*, *SOD1*, *FUS*, *TARDBP*, *NEK1*, *MAPT* and *GRN* mutations. As expected, a significant increase was found in the levels of CHIT1 and YKL-40 in ALS patients carrying any pathogenic mutation. In FTD patients with pathogenic mutations, YKL-40 levels were also elevated, but no subgroup analysis of C9-ALS/FTD patients was reported [242]. A proteomic study confirmed higher CHIT1 levens in C9-ALS versus C9-FTD [243]. A large multicenter study confirmed increased CSF levels of CHIT1 and YKL-40 in FTD patients compared to controls, but C9-carriers were not analysed seperately [244]. In a recent study, we measured CSF biomarkers of neurodegeneration (NfL & pNfH) and inflammation (CHIT1, YKL-40, MCP-1) and correlated them to survival in ALS patients (34,911,782). A multivariate model revealed that the combination of NfL and YKL-40 best fitted the prediction of survival in ALS patients, but we did not observe significant differences in any of the markers between C9-positive and C9-negative patients.

It remains to be seen to what extent the reported changes in inflammatory biomarkers in C9-ALS/FTD are truly caused by the specific mutation, or rather by variability in phenotypic presentations. It could be that there are underlying differences in inflammatory pathomechanisms at play in C9-ALS/FTD, with potentially a disease-specific role of microglia, but more research is needed to further confirm these differences.

### Imaging studies

Several neuroimaging techniques allow to detect and quantify markers of neuroinflammation in vivo. These techniques offer the advantage of measuring markers of inflammation non-invasively in the brain of living subjects.

One such marker of inflammation is the visualization of microglial activation in the CNS through imaging of the 18 kDa translocator protein (TSPO), formerly called peripheral benzodiazepine receptor (PBR) using selective TSPO radioligands. TSPO is a transmembrane cholesterol transporter found primarily in the outer mitochondrial membrane of steroid-synthesizing cells and is thought to be a mediator of cellular metabolic adaptation to various stimuli, including oxidative stress [245]. TSPO is expressed by multiple cell types, including glia and neurons [246]. The protein is suspected to play an important role in inflammatory processes, and overexpression of it is thought to lead to microglial activation [247]. This offered great potential for its use as a visualizer of this mechanism through PET imaging. Over the years, several ligands for TSPO PET imaging have been developed [247–249]. TSPO PET visualization has proved valuable as a biomarker of inflammation associated with several neurodegenerative diseases, including ALS. It may be used as an early biomarker of disease activity and potentially as a marker of disease progression [250].

In ALS, several studies have shown increased TSPO ligand binding in the primary and supplementary motor cortices, pons, frontal lobe, temporal lobe and thalamus [251–258]. There seems to be a good correlation between the level of tracer uptake and the severity of the clinical presentation [255, 258]. In FTD patients, increased uptake of TSPO tracers was seen in frontotemporal regions and in the basal ganglia [259, 260]. Only a few C9-carriers were included in these studies, but there was a trend towards C9-FTD patients having higher 11C-PK-11195 (TSPO ligand) binding [259]. This suggests that neuroinflammation may be more pronounced in C9-FTD, but further studies are required to confirm this.

Another potential tracer to visualize neuroinflammation is the membrane P2X purinoceptor 7 (P2X7) receptor (P2X7R), which showed modestly promising results in ALS patients [257]. No *C9orf72*-specific studies have hitherto been performed.

### Post-mortem studies

Markers of neuroinflammation are clearly present in C9-ALS/FTD brain and spinal cord tissues upon post-mortem neuropathological investigation. The most commonly used markers for microglial activation are cluster of differentiation 68 (CD68), human leukocyte antigen-D related protein-R (HLA-DR), and ionised calcium-binding adapter molecule 1 (Iba1) [261].

In brains of C9-FTLD patients, more dystrophic microglia are found in the cortex of affected brain regions in comparison with healthy controls [262]. Both the number and the morphology (ramified vs. amoeboid) of CD68-positive cells in the frontal and temporal gray and white matter are changed in *C9orf72* [263]*.* A greater abundance of CD68-high microglia is also observed in the white matter. Similarly, a higher number of Iba1-positive microglia is observed in the frontal lobe, particularly the white matter, in comparison to controls. Microglia were more often rod-shaped in C9-FTLD, which indicates an intermediate state between ramified and amoeboid morphology. Microglial dystrophy, which may be a result of impaired lysosomal mechanisms, was also more common across all brain regions than it was in control brains. However, this parameter was highly variable between individuals [261].

When comparing FTLD subgroups of carriers of different pathogenic mutations and those without (known) genetic cause, interesting results have been reported. In one study, GRN-FTLD and C9-FTLD brains were compared. It was demonstrated that C9-FTLD was associated with an increase of CD68–positive microglia in the hippocampus. However, superficial cortical layers in the medial frontal gyrus had greater microvacuolation and greater density of ameboid microglia in GRN-FTLD compared to C9-FTLD. We must note, however, that only TDP-type A-FTLD brains were included in this study, which may not be an accurate representation of *C9orf72* pathology as type A typically represents but a small fraction of *C9orf72* cases [262]. In another study, C9-FTLD brains showed a similar level of microglial activation as non-gene-associated FTLD brains and a lower level than MAPT-FTLD brains [263].

In C9-ALS patients, neuropathologic investigation has shown increased microglial activation in the cervical and lumbar corticospinal tract compared to healthy controls. Intriguingly, a positive correlation between the severity of ALS symptomatology, the lesion load of TDP-43 inclusions and the magnitude of microglial activation was found [264]. The increased microglial reaction using CD68 immunohistochemistry in C9-ALS patients in the pyramidal tract was found to be present at all levels including in the matter underlying the motor cortex, mid-crus cerebri, the medullary pyramids and lateral and anterior corticospinal tracts [265].

When comparing C9-ALS patients with non-C9-ALS patient, one study reported that C9-ALS brains showed more extensive microglial pathology in the white matter of the medulla and the motor cortex (as measured by CD68 and Iba1 immunoreactivity) [265]. Increased CD68 immunoreactivity has also been reported in the body and genu of the corpus callosum of C9-ALS when compared to sporadic ALS [266]. Wheter this inflammatory phenotype is directly linked to the presence of a *C9orf72* HRE or is a consequence of more aggressive disease in general remains unclear.

## Potential anti-inflammatory therapies

There is a high unmet medical need for disease-modifying treatments for FTD and ALS. Riluzole—a glutamatergic transmission inhibitor—and, edaravone—believed to sequester free radicals, ROS and inhibiting oxidative stress—are currently the only FDA-approved therapeutic options for ALS patients [267, 268]. However, they offer only limited effects on disease progression and survival. Modulation of neuroinflammation is a promising way to alter the disease course, in particular in patients with high levels of neuroinflammation, such as C9-ALS/FTD patients. Although a lot of progress has been made in our understanding of the genetic causes and the molecular pathways involved in ALS, there is still a lack of effective therapeutic options due to the heterogeneity of the disease [269].

Several clinical studies have already attempted to diminish inflammation in ALS patients with drugs such as cyclooxygenase–2 (COX) inhibitors, minocycline, sodium chlorite, interferon-beta and thalidomide. So far, no beneficial effects on ALS progression were observed [270]. Little data are available on C9-ALS/FTD patients and no studies have looked at patients with high levels of neuroinflammation separately. In one study (ClinicalTrials.gov Identifier: NCT01277315), anakinra, a recombinant IL-1 receptor antagonist that blocks IL-1β-mediated inflammatory signaling, was used in 19 ALS patients, of which 4 were known to carry a mutation in *C9orf72* gene [271]. The disease progression was measured using ALSFRS-R score and forced expiratory vital capacity as secondary endpoint. IL-6 and TNF-α were also measured as inflammatory biomarkers. However, no correlation between genotype and treatment response was found. Importantly, there was no difference in disease progression, but the IL-6 and TNF-α levels in plasma were also not significantly altered by the treatment. Although IL-1 may serve as a pharmacological inflammatory target for ALS, this study had some limitations such as a small number of patients, not measuring the inflammatory parameters in CSF, and lacking a double-blinded placebo control group.

Nevertheless, C9-ALS patients were shown to have an increased type I interferon signature and a treatment with STING inhibitor suppressed this interferon response which could partially explain the involvement of STING as an underlying mechanism of the enhanced inflammatory profile in C9-ALS patients [222]. Interestingly, small-molecule STING inhibitors showed to decrease the pathology features of autoinflammatory disease in mice [272]. Therefore, different STING inhibitors are proposed as novel, promising therapeutic options once their effectivity and safety in the preclinical development has been demonstrated [273, 274]. There is a surge in the number of trials with anti-inflammatory treatments for ALS/FTD. The current trials specifically for C9-ALS/FTD patients are summarized in Table 1.

**Table 1:**
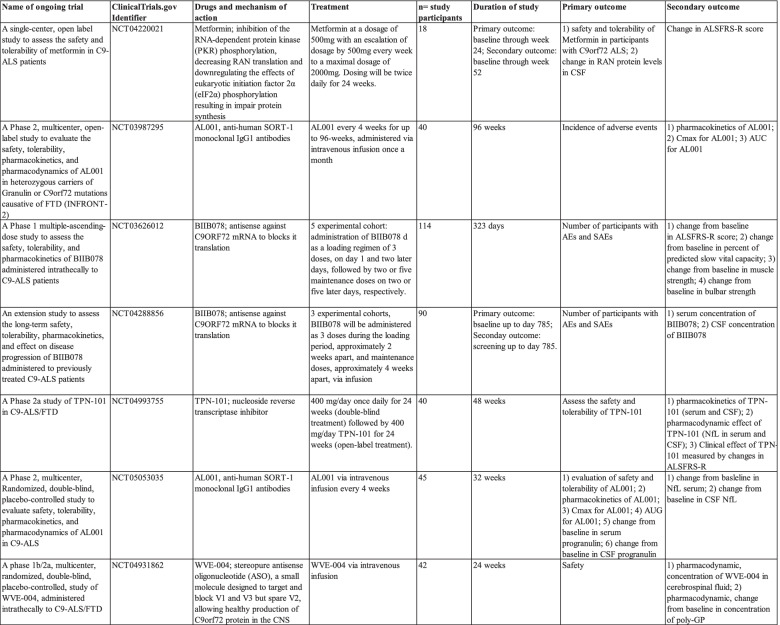
Ongoing clinical trials in C9-ALS/FTD patients

## Conclusion

In this review paper, we outlined the role of neuroinflammation in the pathogenesis of ALS and FTD caused by *C9orf72* HRE. The *C9orf72* gene is highly expressed in innate immune cells and multiple studies demonstrated dysfunction of the immune system caused by *C9orf72* gene mutations. Further studies are needed to clarify the role of neuroinflammation in the initiation and propagation of the neurodegenerative process in C9-ALS/FTD. The crosstalk between the peripheral and CNS immune system also requires further research, as there are many unanswered questions such as: is there an autoimmune component to C9-ALS/FTD? What is the exact role of the inflammatory resonse in the CNS and along the peripheral motor axons? Can changes in the peripheral immune system predict central neuroinflammation and the prognosis of patients? How is the complex process of neuroinflammation contributing to neurotoxicity?

Human biomarker studies focusing on C9-related inflammation are scarce. More specifically, comparisons between sporadic ALS/FTD, C9-ALS/FTD and ALS/FTD without C9 mutations are largely lacking. Although evidence points to a major role for inflammation in the pathogenesis of C9-ALS/FTD, results of clinical trials with anti-inflammatory and immunosuppressive agents have largely been disappointing to date. Early and precise biomarker-based characterization of patients is important in order to identify the subset of patients that are most likely to benefit from anti-inflammatory therapies. Research into identification of reliable disease biomarkers may also aid in measuring the therapeutic effect of novel therapies. Not only wet biomarkers, but also PET imaging may help to identify microglial activation. Robust pharmacodynamic effects on inflammatory biomarkers should be present in order to maximize the chance of success in efficacy trials. Given the role of C9orf72 in the immune system, there is a rationale for anti-inflammatory therapies in C9-ALS/FTD and a better characterization of the neuroinflammatory resonse will hopefully lead to a targetted therapeutic strategy.

## Data Availability

Not applicable.

## Abbreviations

AAD-2004: 2-Hydroxy-5-[2-(4-trifluromethylphenyl)-ethylaminobenzoic acid]
AAV: Adeno-associated virus
ACM: Astrocyte conditioned medium
AD: Alzheimer's disease
ALS: Amyotrophic lateral sclerosis
ALSFRS: ALS Functional Rating Scale
ANG: Angiogenin
ANXA1: Annexin A1
AP-1: Activator protein 1
Arg1: Arginase 1
ASC: Apoptosis associated speck-like protein containing a card
Asymptomatic C9orf72 HREs carriers: Asymp-C9
ATAXN2: Ataxin-2
Aβ: Amyloid beta
BBB: Blood brain barrier
BDNF: Brain-derived neurotrophic factor
bvFTD: Behavioral variant frontotemporal dementia
B-CSF-B: Blood cerebrospinal fluid barrier
CCL2: C–C motif chemokine ligand 2
CCNF: Cyclin F
CCR2: C–C chemokine receptor type 2
CD: Cluster of differentiation
cGAMP: Cyclic GMP–AMP
cGAS: Cyclic GMP–AMP synthase
CHCHD10: Coiled-coil-helix-coiled-coil-helix domain containing 10
CHIT1: Chitotriosidase 1
CHI3L1/YKL-40: Chitinase-3-like-1 protein
Chi3L3: Chitinase-3-like-3 protein
CHMP2b: Chromatin-modifying protein 2b
CNS: Central nervous system
COX-2: Cyclo-oxygenase 2
CSF: Cerebrospinal fluid
CXCL: C-X-C motif ligand
CX3CR1: CX3 chemokine receptor 1
CX3CL1: CX3 chemokine ligand 1
C9orf72: Chromosome 9 open reading frame 72
C9-ALS/FTD: C9orf72 HRE-mediated ALS/FTD
C9orf72 hexanucleotide repeat expansion: C9orf72 HRE
C9orf72 HREs carriers: C9-carriers
DAMP: Damage-associated molecular pattern molecule
DC: Dendritic cell
DENN: Differentially Expressed in Normal and Neoplasia
DPR: Dipeptide repeat
EAAT: Excitatory amino acid transporter
EDN1: Endothelin 1
EEA1: Early endosome antigen 1
EOD: Early onset dementia
ERK: Extracellular signal-regulated kinase
EWS: Ewing’s sarcoma protein
EWSR1: Ewing’s sarcoma protein RNA Binding Protein 1
fALS: Familial ALS
FasL: Fas ligand
FET protein family: FUS, Ewing’s sarcoma protein and TATA-binding protein associated factor 2 N
fFTD: Familial FTD
FOXP3: Forkhead box P3 transcription factor
FTD: Frontotemporal dementia
FTLD: Frontotemporal lobar degeneration
FTLD-FET: FTLD associated with FET pathology
FTLD-FUS: FTLD associated with FUS pathology
FTLD-TAU: FTLD associated with tau pathology
FTLD-TDP: FTLD associated with TDP-43 pathology
FTLD-UPS: FTLD associated with UPS pathology
FUS: Protein fused in sarcoma
GA: Glycine-alanine
GDNF: Glial cell-derived neurotrophic factor
GDP: Guanosine diphosphate
GEF: Guanine nucleotide exchange factor
GFAP: Glial fibrillary acidic protein
GLE1: GLE1 RNA export mediator
GlycA: Glycoprotein acetyl
GP: Glycine-proline
GR: Glycine-arginine
GRN: Progranulin
GTP: Guanosine triphosphate
G-CSF: Granulocyte colony-stimulating factor
HDL: High density lipoprotein
HLA-DR: Human leukocyte antigen-D related protein-R
HMGB1: High mobility group box 1
hnRNPA1: Heterogeneous nuclear ribonucleoproteins A1
hnRNPA2B1: Heterogeneous nuclear ribonucleoproteins A2/B1
HRE: Hexanucleotide repeat expansion
hs-CRP: High sensitivity C-reactive protein
Iba1: Ionised calcium-binding adapter molecule 1
IFN: Interferon
IGF-1: Insulin-like growth factor
IKK: IκB kinase
IL: Interleukin
iMN: Induced motor neuron
iPSC: Induced pluripotent stem cell
iNOS: Inducable nitric oxide synthase
IRAK: Interleukin-1 receptor-associated kinase
IRF: Interferon regulatory factors
JAK: Janus kinase
JNK: C-Jun N-terminal kinase
KIF5A: Kinesin family member 5A
Lamp1: Lysosomal associated membrane protein 1
LCD: Low-complexity domain
LPS: Lipopolysaccharides
lv-PPA: Logopenic variant primary progressive aphasia
MAPK: Mitogen-activated protein kinase
MAPT: Microtubule associated protein tau
MAP2K: Mitogen-activated protein kinase kinase
MAP3K: Mitogen-activated protein kinase kinase kinase
MATR3: Matrin 3
MCP-1: Monocyte chemoattractant protein-1
MHC: Major histocompatibility complex
MIP-1a: Macrophage inflammatory protein 1 alpha
MN: Motor neuron
MND: Motor neuron disease
MRC: Medical research councel
mRNA: Messenger RNA
mTOR: Mammalian target of rapamycin hyjuèy
NADPH: Nicotinamide adenine dinucleotide phosphate
NEK1: Never In Mitosis Gene A-Related Kinase 1
nfv-PPA: Non-fluent variant primary progressive aphasia
NF-κB: Nuclear factor kappa light chain enhancer of activated B cells
NK: Natural killer cell
NKG2D: Natural killer group 2D
NLR: Nucleotide-binding oligomerization domain and leucine-rich repeat-containing receptor
NLRP3: NOD-like receptor family pyrin domain containing 3
NOX2: NADPH oxidase 2
NRF2: Nuclear factor erythroid 2-related factor 2
Nup: Nucleoporin
OPTN: Optineurin
PAMP: Pathogen-associated molecular pattern molecule
PBMC: Peripheral blood mononuclear cell
PBR: Peripheral benzodiazepine receptor
PFN1: Profilin 1
PNS: Peripheral nervous system
PPA: Primary progressive aphasia
PR: Proline-arginine
pTDP-43: Phosphorylated transactive responseDNA binding protein with Mr 43 kDa
P2RX7: P2X purinoceptor 7
p62: Autophagy adaptor protein p62/sequestosome1
RAN: Repeat-associated non-AUG
Ran: Ras-related nuclear protein
RanGAP: RanGTPase-activating protein
RANTES: Regulated upon activation, normal T cell expressed and presumably secreted
RCC1: Regulator of chromosome condensation
ROS: Reactive oxygen species
sALS: Sporadic ALS
SETX: Senataxin
SG: Stress granule
SMCR8: Smith-Magenis Syndrome Chromosome Region, Candidate 8
SOD1: Cu/Zn-binding superoxide dismutase
STAT: Signal transducer and activator of transcription
STING: Stimulator of interferon genes
sv-PPA: Semantic variant primary progressive aphasia
SQSTM1: Sequestosome 1
S1P: Sphingosine 1‐phosphate
S100b: S100 beta
TAB: TGF-beta activated kinase 1 binding protein
TAF15: TATA-binding protein associated factor 2 N
TARDBP: Transactive response DNA-binding protein
TBK1: TANK binding kinase 1
TCR: T cell receptor
TDP-43: Transactive response DNA binding protein with Mr 43 kDa
Teff: Effector T cell
Tfh: Follicular helper T cell
TGF-β: Transforming growth factor beta
Th cell: T helper cell
TIA1: TIA1 cytotoxic granule associated RNA binding protein
TLR4: Toll-like receptor 4
TMEM106B: Transmembrane protein 106B
TNF-α: Tumor necrosis factor-alpha
TRAF6: TNF receptor-associated factor 6
TRAIL: TNF-related apoptosis-inducing ligand
Treg: Regulatory T-cell
TREM2: Triggering receptor expressed on myeloid cells 2
TSPO: 18 KDa translocator protein
TUBA4A: Tubulin alpha 4a
UBQLN2: Ubiquilin 2
UPS: Ubiquitin/proteasome system
VAPB: Vesicle-associated membrane protein-associated protein B and C
VCP: Valosin containing protein
VEGF: Vascular endothelial growth factor
WDR41: WD Repeat domain 41
Ym1: Chitinase-like protein 3
